# Facing Fear Through Making Connections as We Age

**DOI:** 10.1093/geroni/igaa057.2309

**Published:** 2020-12-16

**Authors:** Becky Knight, Amanda Noah, Sharon Bowland

**Affiliations:** 1 University of North Texas, Denton, Texas, United States; 2 University of Tennessee, Knoxville, Denton, Texas, United States

## Abstract

Older residents often experience disruption in family relationships and social networks due to isolation once they move into public housing communities. While the need for social contact continues, the opportunity may diminish. Older African-American women living in one housing community attempted to cope with their personal safety fears through social isolation (Bowland, 2015). Women in the study (N = 25) were survivors of one or more forms of interpersonal trauma, including childhood abuse, sexual assault and domestic violence. In contrast, a few women in the same sample spontaneously formed social networks within the community. This qualitative investigation will examine how the women adapted to an isolating and unsafe environment by developing social connections. As a social determinant of health, developing healthy social networks inside a housing community can reduce fear and improve well-being and quality of life for low-income older residents who are aging-in-place.

